# Ethics of the fiduciary relationship between patient and physician: the case of informed consent

**DOI:** 10.1136/jme-2022-108539

**Published:** 2022-12-23

**Authors:** Sophie Ludewigs, Jonas Narchi, Lukas Kiefer, Eva C Winkler

**Affiliations:** 1 Institute for German and European Corporate and Business Law, Faculty of Law, Heidelberg University, Heidelberg, Germany; 2 National Center of Tumor Diseases, Department of Medical Oncology, Section for Translational Medical Ethics, University Hospital Heidelberg, Heidelberg, Germany

**Keywords:** Informed Consent, Decision Making, Philosophy, Ethics- Medical, Health Personnel

## Abstract

This paper serves two purposes: first, the proposition of an ethical fiduciary theory that substantiates the often-cited assertion that the patient–physician relationship is fiduciary in nature; and second, the application of this theory to the case of informed consent. Patients’ decision-making preferences vary significantly. While some seek fully autonomous decision-making, others prefer to delegate parts of their decision. Therefore, we propose an ethical fiduciary theory that allows physician and patient to jointly determine the physician’s role on a spectrum from fiduciary as advisor to fiduciary as agent. Drawing on legal concepts of the fiduciary relationship and on phenomenological accounts of obligation by Lévinas and Løgstrup, our theory relies on the key attributes of trust, vulnerability and otherness. Finally, practical implications of this theory for the informed consent process are developed: we propose a preassessment of patients’ risk and value profiles as well as a restructuring of the oral consent interview and the written consent materials.

## Introduction

 The fiduciary concept is considered a classic conceptual framework for the patient–physician relationship. Thus, in their famous account of medical ethics, Beauchamp and Childress stated: ‘The patient-physician relationship is a fiduciary relationship—that is, founded on trust or confidence; and the physician is therefore necessarily a trustee for the patient’s medical welfare’.^1^ (p430) According to this general understanding, the main aspects of a fiduciary relationship are personal commitment of the physician and mutual trust. This, however, is far from a fleshed-out theory and it would certainly be difficult to derive specific ethical imperatives from such a broad sense of the fiduciary concept. The question arises whether there is more to be said about the fiduciary nature of the patient–physician relationship and what implications this could have for healthcare practice.

This paper focuses on the ethics of the fiduciary relationship between the physician and the patient: first, in a foundational, theoretical manner and then in the specific context of informed consent. In section 1, the concept of the physician as fiduciary and its legal provenance are introduced. In section 2, the theoretical foundation for the ethical fiduciary concept will be laid. The newly established fiduciary theory is then applied to the specific context of informed consent in section 3. Finally, section 4 draws practical conclusions from the fiduciary theory of informed consent: the most important ones being the introduction of a preassessment in which the patient’s decision-making preferences and values are assessed and the reorganisation of informed consent interviews and the written informed consent documents.

## The legal concept of physician–fiduciary and its implications for an ethical fiduciary theory

Despite the prevalence of the fiduciary concept in medical ethics, its theoretical foundation is still lacking. To better understand the conceptualisation of the physician as fiduciary, we will first turn to the law, where the theory of the physician–fiduciary has been further elaborated.

Historically, courts described relationships in which one person was obliged to act in the best interest of another as relationships of trust. Soon ‘trust’ developed into a distinct legal category pertaining to property. Courts, thus, coined the term ‘fiduciary relationship’ to refer to a broader group of relationships, which did not fit the narrow requirements of the legal trust, but still obliged one person to act for the benefit of another.^2^ Today, fiduciary law governs a myriad of personal, professional and commercial relationships including, for example, the relationship between parent and child, lawyer and client, physician and patient, trustee and beneficiary and corporate director and corporation.^3^ Despite the breadth and importance of fiduciary relationships, courts and commentators have still not agreed on a common definition. Some even believe fiduciary relationships to be indefinable.^2 4 5^ Yet, there is consensus among scholars and the judiciary that all fiduciary relationships share at least the following common attributes: fiduciaries are entrusted with power over the legal or practical interests of another person (the beneficiary). Fiduciaries render services that are socially desirable and require special expertise. To apply their superior knowledge and skill for the benefit of the other, fiduciaries are usually granted discretion. The beneficiary, on the contrary, is vulnerable and dependent on the fiduciary. At the heart of this asymmetric relationship lies the special trust that the beneficiary reposes in the fiduciary. To protect the trust and integrity of the relationship, fiduciaries are held to the highest standard of conduct: They owe the beneficiary “[n]ot honesty alone, but the punctilio of an honor the most sensitive”.^6^ (p464) Accordingly, fiduciaries are bound by a duty of care, loyalty and good faith.^7^,^14^

The patient-physician relationship fits this description well: the patient usually lacks medical expertise, making them dependent on the physician’s knowledge and judgement. The patient entrusts the physician with power over their body and grants them discretion with regards to their medical judgement. The hallmark of the patient–physician relationships is the special trust, which patients place in their physician.^1015^,^21^ As the Supreme Court of New Jersey elaborated: ‘Few decisions bespeak greater trust and confidence than the decision of a patient to proceed with surgery. Implicit in that decision is a willingness of the patient to put their life in the hands of a known and trusted medical doctor’.^22^ Similarly, the Supreme Court of Canada found it ‘readily apparent that the doctor-patient relationship shares the peculiar hallmark of the fiduciary relationship’.^23^ Today, courts and commentators almost unanimously acknowledge the fiduciary nature of the patient–physician relationship.^20 21^

To ensure the full and accurate application of fiduciary principles in medical contexts, it is vital to clarify a common misconception: physicians are fiduciaries for their patients; however, they are not trustees in the strict sense of trust law, as indicated by some sources.^1^ Trustees are entrusted with property. Physicians are entrusted with power over the patient’s body—which can hardly be qualified as property. The patient–physician relationship falls into the broader category of the fiduciary relationship, but not into the narrow proprietary subgroup of the trust. The depiction of the physician as trustee causes confusion and, in the worst case, results in an inappropriate application of trust law, which does not fit the requirements of the patient–physician relationship.^24^

Based on the legal concept of the physician as fiduciary, Chervenak, McCullough and others have developed first ethical accounts of the physician as fiduciary: in medical ethics, ‘[t]he concept of the physician as fiduciary means that the physician (1) is an authority—that is, they possess expert knowledge and skills on how to protect and promote the health-related interests of the patient, and (2) are committed to using that expertise primarily for the benefit of the patient and to making self-interest a systematically secondary condition’.^25^ (p174)

It is the purpose of this paper to show how such legal aspects and first ethical accounts of the fiduciary concept can be grounded in a deeper, phenomenological analysis of ethical obligation and then be applied to the case of informed consent. In the informed consent process, the patient entrusts the physician with a host of responsibilities: (1) inquiring into and seeking to understand their personal values, (2) evaluating which information is necessary for a sufficiently informed decision according to these values, (3) framing the information accordingly and (4) (if wanted) making recommendations based on this knowledge. We call this ‘fiduciary informed consent’.

## An ethical foundation of the fiduciary relationship between patient and physician

In previous ethical accounts of the fiduciary concept, commentators have often simply assumed the fiduciary nature of the patient–physician relationship without providing a theoretical foundation for it. If at all, the fiduciary character has been justified by reference to either a long historical tradition^26 27^ or the use of the concept in law. While these contexts are important to understand the genesis of the concept, they have a rather limited importance for its validity. The question remains: What are valid reasons to consider the patient-physician relationship fiduciary in nature? To answer this, the fundamental structure of ethical obligation needs to be investigated. This will uncover an ethical substructure underlying intersubjective interactions of trust that may serve as a starting point for rethinking the fiduciary relationship between patient and physician.

Generally speaking, there are two views concerning the source of ethical obligation: In the words of Christensen, ‘individualistic’ approaches—such as Immanuel Kant’s—place the source of obligation within the individual, while ‘relational’ views place it outside the individual, that is, in the other person or, more precisely in their relationship to one another.^28^ Among the authors Christensen cites in support of the latter view, two in particular merit to be analysed here: The Lithuanian-French philosopher Emmanuel Lévinas (d. 1995) and the less known Danish philosopher and theologian Knud E. Løgstrup (d. 1981). As will be shown, their relational accounts will offer a starting point for an ethical fiduciary theory precisely because of their focus on interpersonal relations as the ground of ethical obligation.

In his ground-breaking book *Totality and Infinity*, Lévinas analysed the phenomenological structure of intersubjectivity in general and the ethical structure of obligation in particular. According to his analysis, the ethical substructure of intersubjective relations is that of the *self* and the *other* that is mediated by the *face*, a term that encompasses all aspects of how a person appears. The *face* reveals the *otherness of the other* who is fundamentally vulnerable, irreplaceable and always eludes the self to some extent. The standpoint of the other is never fully interchangeable with one’s own because the freedom of the self encounters the vulnerability of the other as its boundary. ‘But the […] absolutely other […] does not limit the freedom of the same; calling it to responsibility, it founds it and justifies it’.^29^ (p197) Out of the freedom of the other arises, the ethical obligation to respect them and to take responsibility for them without taking away their freedom.

Applying Levinas’ phenomenology to the field of medical ethics, it has been proposed to think of the patient–physician relationship as a special manifestation of the relation of the self and the other. Compared with Levinas’ general account of the self and the other, the physician–patient relationship is ‘qualified’ in two regards. As healthcare professionals, physicians already bear a general responsibility for any potential patient resulting in a special duty to aid in cases of emergency. When the patient entrusts themselves in the care of the specific physician and the physician accepts this entrustment, the physician’s general responsibility towards any potential patient becomes a specific obligation towards this individual patient.

In the ‘face of the patient’, there appears a fundamental vulnerability and non-interchangeable freedom that obliges the physician to take responsibility for them while, at the same time, respecting their otherness^30^: The patient’s freedom and vulnerability provide the foundation for the freedom and responsibility of the physician to care for them. Benito and García have applied Lévinas’ theory to informed consent: ‘In the process of IC [informed consent] […] the patient’s Face appears: this Face demands support and protection and limits patient’s autonomy. Consequently, the physician is asked by the patient to take responsibility for the Other. […] *Good medical practice* has to incorporate a patient-centered responsibility, a protection that respects the autonomy of the subject and does not allow indifference’.^31^ (p452) Accordingly, they argue for a shift from an informed consent concept solely based on the principle of autonomy to an account that includes beneficence and non-maleficence to a higher degree: faced with the otherness and vulnerability of the patient, the physician should not rely solely on the patient’s wish, but consider whether an intervention is truly beneficent or, in the words of fiduciary theory, in the patient’s best interest. From the perspective of fiduciary theory, the asymmetric relationship of the self and the other is best described—not as paternalistic on one end of the spectrum or as solely based on the patient’s autonomy on the other—but as an intermediary one, in which physician and patient share the responsibility for the patient’s health.

As has been pointed out by Christensen, Lévinas provides a comprehensive account of the structure of ethical obligation, however, he ‘does not present a theory about the content of ethical obligation’.^28^ (p25) For this, we turn to Løgstrup’s account. In *The Ethical Demand*, Løgstrup chooses a similar approach grounded in the phenomenological analysis of the relationship of trust. He does, however, differ from Lévinas in one important detail: while Lévinas sees the source of ethical obligation solely in the otherness of the other, Løgstrup maintains that ‘ethical responsibility arises […] from the nature of the relationship itself, the trust between human beings’.^28^ (p30) Løgstrup starts his argument with the observation that human existence is grounded in trust: ‘It is integral to human life that we normally meet each other with natural trust’.^32^ (p9) Even strangers are met with trust. Distrust only arises when there is reasonable doubt that the other can be trusted. Trust, however, entails the possibility of hurt, because ‘to show trust is to deliver oneself up’.^32^ (p10) In trusting the other, for example, to show me the way to a train station I cannot find, I expose a vulnerability and lack of knowledge while, at the same time, causing a demand that can be met or disregarded by the other. ‘Regardless of how varied the communication between us may be, it always consists in daring to come forward to be met by the other. This is […] the basic phenomenon of ethical life. Therefore, the demand that arises from this needs no revelation in the theological sense […], and nor does it come from a […] conscious arrangement’.^32^ (p17) The demand arising from a relationship of trust is indeed unspoken and can even contradict the spoken request of the person: ‘The other human being’s own interpretation of what the trust they show or desire is really about is one thing; the demand which is implicit in that trust […] is quite another thing’.^32^ (p20) In other words, there is a potential conflict between the self and the other and the way they interpret the ethical obligation arising from their relationship of trust. Paradoxically, such a conflict is not detrimental to the relationship but instead a sign of freedom: ‘If this were not the case, a communication between us—on a basic and existential level—[…] would not be possible. For if it were merely a matter of responding to the expectation of the other and fulfilling their wish, our life together would simply consist in—irresponsibly—making ourselves into the tool of the other person’.^32^ (p20)

Considering these phenomenological accounts of obligation as an ethical relation between the self and the other, the fiduciary relationship between patient and physician appears as a special manifestation of it: by showing trust, patients entrust themselves to the physician adding the vulnerability of trust to the already existing health-related vulnerability. Since the establishment of this relationship, there has been an unspoken demand extending from the other, the patient, to the self, the physician, (or vice versa) that the one shall respect and protect the other. The same relationship might be viewed as hierarchical from either perspective: because of a special vulnerability, the patient is ‘at the hands’ of the physician who seems to be in power. At the same time, the patient imposes a fundamental ethical imperative on the physician that Lévinas has described as the face of the other that cannot be replaced. To understand the patient–physician relationship as fiduciary, it is to find a middle ground where the self and the other meet at eye level. In Løgstrup’s words: ‘From this fundamental dependence […], the demand arises that we take care of that in the other person’s life which is dependent upon us, and which we have in our power. However, based on the same demand, it is forbidden that we ever attempt to rob the other person of their independence, even for their own sake. Responsibility for the other person can never consist in our taking on the responsibility which is their own’.^32^ (p26) Because of the divide of otherness, neither the patient’s nor the physician’s freedom can be absorbed by the other. Thus, the fiduciary model navigates the middle ground between the paternalistic model on the one and the consumer model on the other end of the spectrum.^33^ To say that the physician as fiduciary makes the patient’s interests their own, therefore, is not to say that the physician yields all judgement to the patient (nor the other way around). Instead, physician and patient need to find and evaluate the convergence or divergence between the spoken demand of the patient and the unspoken demand of the situation. How this could be done in the case of informed consent, shall be outlined in the following.

## The theory of fiduciary informed consent

Moving from the abstract ethical theory of the fiduciary relationship to a more concrete application of it, it shall be shown that the matters of trust and obligation detailed with the help of Levinas and Løgstrup resurface in the legal and ethical discussion of informed consent: In American law, the informed consent doctrine is regarded as ‘the most direct application of fiduciary principles’.^21^ (p295) Since the introduction of the patient-centred informed consent doctrine in *Cobbs v. Grant* and *Canterbury v. Spence*, courts have relied heavily on the physician’s fiduciary status to explain their duty to inform the patient.^16 21^ With regards to the patient’s lack of medical information, the hallmarks of the fiduciary relation—the patient’s vulnerability, their trust and reliance on the physician—become apparent. Accordingly, the California Supreme Court reasoned in *Cobbs* that ‘the patient, being unlearned in medical sciences, has an abject dependence upon and trust in his physician for the information upon which he relies during the decisional process, thus raising an obligation in the physician that transcends arms-length transactions’.^34^ In *Canterbury*, the court explicitly identified these ‘fiducial qualities’ as the basis of the ‘physician’s duty to reveal to the patient that which in his best interests it is important that he should know’.^35^

### Fiducial qualities relevant for informed consent

With the help of Levinas and Løgstrup, we have been able to demonstrate that the ethical obligations between physician and patient rely precisely on the attributes which the courts have identified as the ‘fiducial qualities’: vulnerability, trust and otherness. But how do these qualities relate to the issue of informed consent?

1. *Vulnerability*: the fiduciary relationship is asymmetric in so far as the physician is in a position of superiority, while the patient is vulnerable; first, because of poor health, second, because of their lack of expertise. In the context of informed consent, the patient’s vulnerability has the following implications: first, the psychophysical vulnerability is exposed in the disease and the need for care. Patients are often willing to do whatever it takes to receive the interventions that promise to increase quality or length of life. Second, the patient’s vulnerability takes the form of lack of knowledge, which renders the information process necessary. Regarding informed consent, the patient–physician relationship is, therefore, particularly asymmetrical, which further enhances the patient’s reliance on the physician.
2. *Trust*: out of the trust between the patient and physician arises an ethical obligation for both of them (but especially for the latter) to weigh alternative courses of action and to find the unspoken demand of the situation. To identify the unspoken demand in the context of informed consent, the patient and the physician must collaboratively assess the risks of the treatment and identify the patient’s affected values. Medical decisions are often value-depended. Yet, patients rarely know about the importance values bear on medical decision-making. They might not even have a spontaneous answer as to what their values or preferences are. Patient’s values and preferences are, thus, often ‘unspoken demands’. The unspoken demand might not always be identical with the spoken demand: for example, some patients’ spoken demand might be not to learn about serious risks of a treatment, yet the unspoken demand of the situation may still require the physician to inform them about such risks. It is vital to note that this is not a paternalistic position. It does not allow the physician to ever over-ride the patient’s decision. ‘Identifying the unspoken demand’ in the context of informed consent simply means to identify the patient’s values and preferences.
3. *Otherness*: due to the otherness of the other who can never be substituted, it is ethically forbidden for one to completely substitute the responsibility of the other. Both, the paternalistic substitution of the patient’s will with the physician’s and (for the lack of a better term) the ‘autonomist’ substitution of the physician’s will with the patient’s, are to be avoided. Instead, patient and physician need to work out their distinct responsibilities in a given situation and the extent to which decisions can be delegated (and the extent to which the patient is willing to do so). Applying this to informed consent, the following can be derived: due to the otherness of the patient (and vice versa of the physician), it is ethically problematic to completely substitute the other’s decision-making capacity. There surely is a right not to know as well as there is a right to know, but it is not unlimited. There will always be parts of a decision that cannot be fully delegated and must be discussed with the patient. On the other hand, there are parts that may well be delegated to the physician, should the patient choose to do so. Therein lies, as will be shown below, the significance of a preassessment of the patients’ preferences and values and an agreement between patient and physician on the extent to which the physician is allowed to act in the patient’s interest.

### Theoretical implications for a framework for fiduciary informed consent

How can these implications be translated into a more elaborate theory of fiduciary informed consent? In 2009, Joffe and Truog laid the foundation for such a theory that can serve as a starting point: their account begins with the assertion that informed consent, understood as the process in which patients autonomously authorise medical interventions, is fundamental to ethical practice in healthcare. At the same time, in everyday life, patients often claim decision-making responsibility only to a limited extent and entrust physicians with a substantial part of the decision. According to the authors, the fiduciary concept as a solution to this problem can serve two different purposes: it can conceptualise an existing practice in a *descriptive* manner and *normatively* regulate such practices.^36^ How can it do so in the case of informed consent?

Due to different factors among which time and resources might play an important role, physicians often do not live up to their obligation to facilitate informed consent and do not present the patient with all treatment alternatives, risks and benefits, but instead propose only the intervention they deem best. The result could be called ‘minimally informed acquiescence rather than true informed consent’.^36^ (p347) This, however, seems to be accepted or even desired by many patients who prefer a reserved decision-making role. A model that could explain the implicit responsibility transfer that occurs in these situations is the fiduciary model: using a legal analogy derived from Shepherd,^37^ Joffe and Truog describe two relevant roles a fiduciary can play: the fiduciary *as agent* acts on behalf of their client without requiring specific authorisation for individual actions. Rather, this autonomy is given to them by the client on entering into the fiduciary relationship. The fiduciary *as adviser* primarily provides the client with information and is never authorised to act as their representative without the client’s consent.^36^

It is easy to see that neither of these conceptualisations exclusively accounts for the complexity of the patient–physician relationship: the concept of a fiduciary as agent is only appropriate if the patient is unable to take on the role as a decision-maker or has fully delegated this to the physician. Also, physicians cannot know the patient’s values and preferences in advance to make important medical decisions as their representatives. Conversely, the fiduciary as adviser would not be realistically applicable to complex medical treatments that require the physician to make numerous minor decisions without always being able to obtain the patient’s explicit consent.^36^ The authors, therefore, advocate for a mixed model: mirroring the middle ground between the self and the other that we have outlined in light of Levinas’ and Løgstrup’s phenomenological analysis, they propose ‘that every interaction involving a physician and a competent adult patient inevitably straddles the agency and advisor models of fiduciary relationships’.^36^ (p355) These models should be thought of as a spectrum where ‘different physician–patient dyads occupy different points on the continuum between these two archetypal relationships’,^36^ (p355) providing the opportunity for degrees of delegation depending on the patient’s individual preferences. In other words, the fiduciary framework is no one-size-fits-all-solution but depends on the patient’s autonomous will to form their informed consent according to their own requirements. It will be shown below, how this aspect of the mixed fiduciary model can be brought into practice by introducing a preassessment of the patient’s preferences.

Now, how can the mixed model be applied to specific situations of the informed consent process? Which ‘parts’ of medical decisions can be delegated to the physician as agent and where do they function merely as advisors? Joffe and Truog propose the distinction between choices about ends and choices about means: ‘Patients’ values inform decisions about ends, whereas once patients and physicians reach agreement about ends, technical considerations that lie within the domain of medical expertise inform decisions about means’.^36^ (p356) Therefore, generally speaking, choices about ends lie within the domain of the patient and the physician-fiduciary as advisor, while choices about means lie within the domain of the physician-fiduciary as agent and the patient as beneficiary. To cite an example in the context of end-of-life decision-making, the choice between longevity and quality of life is a choice of ends highly dependent on patients’ values, while the choice between two different anaesthetics is a choice of means that seems almost value-neutral. Therefore, the former decision cannot be delegated to a physician–fiduciary whereas the latter can. It is not always clear to what extent a decision primarily concerns ends or means (especially since these are often intertwined), but according to the authors, they, too, can be located on a spectrum. On this spectrum, the patient’s values and preferences are more relevant, the more a decision affects the ends of the treatment.

Additionally, there can be cases in which the choice of means is value dependent, thereby necessitating the patient’s consent. To take a different example than the one of breast cancers cited by Joffe and Truog, blood transfusions seem to be a neutral means during an operation. Yet, religious groups such as Jehovah’s Witnesses oppose blood transfusions as contrary to divine law. Accordingly, the decision (whether to do a blood transfusion) becomes value-dependent as it touches on the patient’s religious beliefs.^38^ Similarly, porcine medicinal products could be value-dependent means as potentially violating Jewish or Muslim dietary rules, while bovine products could be in conflict with Hindu faiths.^39^ This illustrates the importance of establishing a patient’s value profile because it shows how complex the spectrum can be. Covering the beginning, middle and end of the means-ends-spectrum, Joffe and Truog end with the following hypothesis:

1. ‘Patients are always responsible for medical decisions about the ultimate goals of therapy, which necessarily involve weighing of values.
2. Patients are presumptively responsible for decisions about the means to those ends, to the extent that such decisions entail value-laden choices among subsidiary ends.
3. Physicians may assume presumptive responsibility for those decisions about means that are unlikely to entail value-laden choices between subsidiary ends’.^36^ (p360)

Conceptualising informed consent in a fiduciary framework, simultaneously allows for a greater and lesser degree of delegation and autonomous decision-making on the patient’s part depending on their decision-making preference and value profile. Far from being a return to paternalism in a new form, the fiduciary concept empowers patients to decide for themselves to what extent they want to exercise their autonomous decision-making capacity and whether they want to delegate certain parts of the decision to the physician as fiduciary. The fiduciary framework encompasses both a right to know and, to a certain degree, a ‘right not to know’. The latter, however, is limited: some decisions cannot be delegated as their delegation would undermine the patient’s autonomy and violate the physician’s by entrusting decisions to them that are outside their responsibility.

## Practical consequences of fiduciary informed consent

Fiduciary informed consent relies on the rationale that patients differ in their preferred model of decision-making and the roles they assign the physician: while some patients seek fully autonomous decision-making (physician as advisor), others prefer shared decision-making (deliberative model) and again others prefer to delegate parts of their decision (physician as agent).^33^ If self-determination is taken seriously as justification and goal of informed consent, the informed consent process must be tailored to the patient’s individual preferences. These theoretical implications have direct practical consequences: first, the introduction of a preliminary discussion where the patient’s decision-making preference and value profile are assessed, and second, a reorganisation of the consent interview and the written consent materials.

### Preassessment of patients’ preferred role in decision-making

The first goal of the preliminary discussion is to determine the patient’s desired role in the decision-making process. If the patient prefers an active role, the physician serves only as an advisor, merely providing the necessary information to enable the patient’s independent decision-making. If the patient desires collaborative decision-making or a reliant role, the physician becomes more of an agent. As agent, the physician not only provides information but also offers concrete suggestions.

For the development of patient profiles with regards to their informational preferences, the fiduciary concept can use results from the ongoing empirical research on informed consent in healthcare practice. In a study on patients’ attitudes towards informed consent for anaesthesia and surgery, Burkle *et al* found that while most patients (61%) felt that the benefit of information outweighs the negative effects produced by it, 21% of patients believed the opposite. Of the former information-affine group, 80% wanted the disclosure of rare but severe risks and nearly all of them (97%) agreed with the disclosure of common but less severe risks. The proportion of patients who want disclosure is significantly smaller in the group that can be characterised as information-averse (66% and 80%, respectively). This group can be interpreted to be more willing to take risks and delegate control to the physician.^40^ There seem to be distinct information and risk types on a spectrum from those demanding more information and being less ready to take risks to those rather information-averse and more willing to take risks.

With these results as a starting point for a roughly sketched typology, three patient information and risk types could be distinguished: group A, on one end of the spectrum, can be described as highly information-seeking and risk-averse, while group C, on the other end of the spectrum, is more risk-taking and information-averse. Group A prefers the physician–fiduciary as advisor, whereas group C tends towards the physician–fiduciary as agent. Group B includes patients ‘in between’ who are willing to delegate parts of the decision-making process while reserving others for their own judgement. With regards to group B, the physician takes on a mixed fiduciary role. The physician’s role is, therefore, not identical in all patient–physician relationships. Rather, it depends profoundly on the patient’s individual preferences. These classifications are no static, one-time decisions between physicians and patients but remain subject to the autonomy of the patient and can, therefore, change with their situation. Changes in patients’ information preferences may lead to a re-evaluation of their risk type and a subsequent change to the fiduciary role of the physician.

The assessment of the patient’s risk type should be combined with an assessment of the frequency and severity of complications which might occur during the intervention. According to Carlisle, aspects with a high severity of harm should be disclosed even if the likelihood of the risk’s occurrence is low. Those with little severity of harm do not always need to be disclosed even if they are more likely to occur.^41^ We propose that the risk type of the patient should be matched to this, so that a risk-averse patient is informed about all potential risks of an intervention regardless of their severity of harm (but still an order that reflects the severity, see below), while more risk-taking patients need only be informed about the most harmful risks.

Another way to approach the question would be to classify patient risk and information types with regards to their role in the shared decision-making process by employing the Control Preference Scale developed by Degner *et al*.^42^ In cross-sectional interviews in end-of-life situations, the researchers distinguished five groups of patients according to their control preferences ranging from ‘I prefer to make the decisions about which tests or treatments I receive’ to ‘I prefer to leave all decisions about which tests or treatments I receive to my doctor’. These five groups were later clustered into three general classifications: ‘active’, ‘collaborative’ and ‘passive’. In our categories introduced above, group A would be independent, group B would seek shared control and group C would be reliant on the physician as fiduciary agent. This example illustrates how well shared decision-making concepts fit into the fiduciary theory, providing an opportunity for a combination of shared decision-making research and fiduciary theory in ethics and law.

### Preassessment of patient value profiles

The second goal of the preliminary conversation is to encourage the patient to reflect on personal values that may determine what they deem appropriate to delegate. As the discussion of Joffe and Truog has shown, whether or not information must be disclosed and decisions can be delegated, depends not only on potential risks and harms but also on the values a decision might touch on. Decisions concerning the ends of a medical intervention are always value-based and cannot be delegated. They must always be discussed with the patient even if the patient identifies as a group C patient. Regarding decisions about means, the matter is more complicated: the religious examples cited above have shown that means can be value laden. Accordingly, medical means, which otherwise do not need to be discussed, might become an essential part of the informed consent process due to the patient’s specific values. The preliminary conversation should convey that medical decisions can be value-laden and that value-laden decisions must not be delegated. Since specific value-laden decisions that might arise during future consent interviews cannot be anticipated, the preliminary conversation should only address in a general manner what is most important to the patient. Such a general understanding of the patient’s most important values serves as a starting point for the discussion of specific value-laden decisions in future consent interviews and provides the patient with an impetus to reflect on their personal values ahead of time.

### Preassessments within the constraints of the clinical environment

Given the limited resources in the clinical environment, the introduction of additional preassessments must be carefully considered to avoid putting yet another burden on already strained physicians. Preassessments have proven to significantly improve the quality of informed consent and likely save time in the long run. While decision-making preferences and values become more important in high-risk interventions, if preferences among patients vary considerably, or if there is no consensus on the standard of care within the medical profession,^43^ conveying the rationale—that personal values and decision-making preferences matter—positively impacts all medical decisions and treatment relationships. Especially if patients have not previously considered their preferences and values, presenting them with the importance of personal values for medical decisions and possible roles in the decision-making process promotes a better understanding of the medical decisions they are facing, helps develop clearer treatment preferences and encourages patients to take on a more active role.^42 44^

While the benefit of reaching closer to the ideal of full self-determination is a strong argument for the introduction of preassessments, counterarguments regarding feasibility must not be disregarded. Preassessments do require more time. But they are not as resource-consuming as one might assume: First, the preliminary conversation is conducted only once when the patient–physician relationship is first created. The assessment is recorded for all future interactions and will be made available to the treating physician on every subsequent visit. Preferences can always be amended, but the full upfront assessment will not have to be repeated each time. Second, even the upfront assessment should take no longer than a couple of minutes. The preliminary conversation is designed as a brief discussion on the importance of values and decision-making preferences that must be ‘high level’ enough to inform all subsequent interactions with the patients. Medical decisions that arise during the course of a treatment relationship vary in nature and significance. ‘High-level parameters’ can be applied to different medical decisions and can be enriched with further information during the respective consent interview. Further research must show whether the preliminary conversation is best conducted as an open discussion between patient and physician or whether standardised preference assessment tools should be introduced. Standardised preference assessment tools have been appraised for their ease of interpreting, administering and completing within a reasonable time and without prior training in previous studies.^45^ But further studies are needed to confirm these results in the clinical routine.

Regardless of the concrete method, institutionalised preassessments provide two considerable benefits: they significantly improve the quality of informed consent and take pressure off the physicians as they would neither be expected to simply ‘know’ what is important to the patient nor have to justify taking additional time to inquire into such values.

### Restructuring the oral informed consent discussion

The preassessment is only the first step to achieving fiduciary informed consent. In a second step, the consent interview must be adapted to the patient’s decision-making preferences and values, which have been identified in the preliminary conversation. The consent interview should distinguish between decisions about ends and decisions about means. When discussing means, the physician should highlight all value-laden questions the particular case might prompt, for instance the use of bovine products that interfere with the patient’s religious beliefs. Such a distinction by means and ends provides a logical structure for the consent interview as decisions about ends logically precede decisions about means. More importantly, the distinction clarifies which decisions are value laden in the particular case, serving as a reminder that these decisions must not be delegated and should only be made after careful consideration of the patient’s values.

### Restructuring of written informed consent material

As a further practical consequence, the written information material must be reorganised into three sections:

The first section should contain general information about ends. Patient information sheets regularly refer to a specific treatment—hence only to specific means. Nevertheless, all patient information sheets should include a brief first section with abstract questions about ends to remind the physician that the consent interview should begin with a discussion of ends.

The second section should pertain to value-laden decisions about means. While standardised consent materials cannot encompass information pertaining to every idiosyncratic belief the information sheet should highlight decisions that could conflict with commonly held beliefs, such as the use of animal products that violate common (religious) dietary rules.

The third section should discuss value-neutral decisions about means, stating information about the risks of the specific treatment. Instead of providing the information according to medical criteria such as the order of affected organs or the consecutive steps of the intervention, an order of *relevance* should be employed. The risks highest in severity of harm and likelihood of occurrence should be described first, less severe or less likely risks should follow.

Organising the written consent material in the proposed order allows physician and patients to easily access the information the patient seeks according to their individual preferences while also ensuring that all patients receive at least the required information. Disclosures about ends and value-laden means are required as such decisions can never be delegated to the physician without violating the patient’s autonomy. Similarly, disclosures about the most common and most severe risks are compulsory because self-determination requires an *informed* consent. To make an informed decision, the patient must, at a minimum, understand the risks highest in severity and likelihood. Group C patients would only need to read the first page(s): section 1 about ends, section 2 on value-laden means and the beginning of section 3 on the most common and severe risks. If desired, they could stop there and would be spared from information overload. Patients who choose to gather further information could read the remaining pages on risks of lesser severity or likelihood. Such restructured information sheets guarantee that the extent to which patients are willing to entrust their responsibility to the physician remains within their autonomous decision.

## Conclusion

Starting with the legal classification of the patient–physician relationship as fiduciary, we have demonstrated that the fiduciary concept can provide normative guidance for the case of informed consent once it is founded on a deeper ethical theory of fiduciary obligation. Drawing on phenomenological accounts of obligation by Lévinas and Løgstrup, our ethical fiduciary theory relies on the key attributes of vulnerability, trust and otherness: because of their lack of medical expertise and the trust they repose in the physician patients are vulnerable, they are dependent on the physician. From this dependence stems the physician’s obligation to take care of the patient and to act in their best interest. Yet, the otherness of the patient does not allow the physician to ever undermine the patient’s independence, not even for their own sake. The fiduciary nature of the patient–physician relationship forbids a paternalistic substitution of the patient’s will as much as it forbids the physician to yield all judgement to the patient. Instead, the fiduciary theory navigates the middle ground between the paternalistic model on the one and the consumer model on the other end of the spectrum; it allows physician and patient to share the responsibility for the patient’s health.

Combining our ethical theory of the fiduciary patient–physician relationship with informed consent theories, several practical implications can be drawn: the degree to which responsibility can be delegated to the physician in the informed consent process depends on the patient’s willingness to do so and on the individual relationship of trust between physician and patient. Therefore, we propose the introduction of a preassessment of the patient’s information, risk and value type to enable the patient to choose how the physician should act on the spectrum from fiduciary as agent to fiduciary as adviser. Based on this individual assessment, the physician can tailor the informed consent process to the patient’s individual needs. To facilitate such personalisation, the consent interview should distinguish between decisions about ends and means and highlight all value-laden decisions. Accordingly, the written consent material should be reorganised into three distinct sections: section 1 should contain basic information about ends; section 2 should highlight value-laden decisions about means; section 3 should pertain to value-neutral decisions about means, stating information about the risks of the specific treatment beginning with the most common and severe risks. Our fiduciary theory is not limited to informed consent. In our opinion, the answer to many of the ethical questions in healthcare could lie in the often cited but seldom explained assertion that the patient–physician relationship is fiduciary in nature.

## Data Availability

Data sharing not applicable as no datasets generated and/or analysed for this study.
